# Cardiac performance, biomarkers and gene expression studies in previously sedentary men participating in half-marathon training

**DOI:** 10.1186/2052-1847-6-6

**Published:** 2014-02-19

**Authors:** Danica D Vance, Gordon L Chen, Mark Stoutenberg, Robert J Myerburg, Kevin Jacobs, Lubov Nathanson, Arlette Perry, David Seo, Pascal J Goldschmidt-Clermont, Evadnie Rampersaud

**Affiliations:** 1 John P. Hussman Institute for Human Genomics, University of Miami, Miami, FL, USA; 2 Department of Kinesiology and Sports Science, University of Miami, Miami, FL, USA; 3 Department of Medicine, Miller School of Medicine, University of Miami, Miami, FL, USA; 4 Department of Physiology, Miller School of Medicine, University of Miami, Miami, FL, USA; 5 Dr. John T. Macdonald Foundation Department of Human Genetics, University of Miami, Miami, FL, USA; 6 Department of Epidemiology & Public Health, Miller School of Medicine, University of Miami, Miami, FL, USA

**Keywords:** Gene expression, Exercise, Cardiovascular, Response

## Abstract

**Background:**

The mechanisms through which exercise reduces cardiovascular disease are not fully understood. We used echocardiograms, cardiac biomarkers and gene expression to investigate cardiovascular effects associated with exercise training.

**Methods:**

Nineteen sedentary men (22–37 years) completed a 17-week half-marathon training program. Serial measurements of resting heart rate, blood pressure, maximum oxygen consumption, lipids, C-reactive protein, cardiac troponin T, echocardiograms and blood for gene expression were obtained from baseline to peak training. Controls included 22 sedentary men who did not exercise.

**Results:**

Among the training group, VO_2_ max increased from 37.1 to 42.0 ml/kg/min (p < 0.001). Significant changes were seen in left ventricular wall thickness and mass, stroke volume, resting heart rate and blood pressure (p < 0.001). The control group demonstrated no significant changes. Expression profiling in the training group identified 10 significantly over-expressed and 53 significantly under-expressed loci involved in inflammatory pathways. Dividing the training group into high and low responders based on percent change in VO_2_ max identified loci that differentiated these two groups at baseline and after training.

**Conclusion:**

Intensive exercise training leads to significant increase in cardiac and hemodynamic performance, and significant changes in expression of genes involved in immune and inflammatory response.

## Background

Long-term exercise training has been associated with weight loss, improvements in lipid profile and blood pressure, and cardiac remodeling [1-3]. Despite this, the cardiovascular response to high-intensity training in previously untrained individuals is not well characterized. Importantly, even with extreme marathon and half-marathon (HM) exercise training, individuals may experience varying levels of cardiovascular and physiological changes, and these differences may have a biological basis. For example, HM and marathon training have been associated with increases in oxidative stress and myocardial damage [4-9]. HM and marathon training also induces the release of troponin I and T, markers of myocardial injury; elevations in cardiac troponin T (cTnT) are reported in up to 39-56% of athletes immediately after performance [10]. Although the mechanism and implications are not well defined, these biomarkers usually return to normal within 24 to 48 hours, and the magnitude of biomarker peak is inversely related to the level of training prior to the event [10]. The purpose of our study was to assess time-related cardiovascular performance and function and corresponding gene expression changes in participants undergoing HM training. Our goal is to begin to identify a genomic basis for response to exercise by correlating specific changes in gene expression with the physiologic changes following HM training.

## Methods

Apparently healthy, untrained and sedentary males (n = 47), between 18 and 45 years of age, participated in the study. Candidates were required to have engaged in less than 2 hours of aerobic training per week prior to enrollment, have an average maximum oxygen consumption (VO_2_ max) less than 50 ml/kg/min, and not previously completed a HM. Twenty-four of the subjects, who were enrolled in a local HM Training Program, were recruited into the training group (TRAIN), and twenty-three male subjects of similar age were recruited into the control group (CON). The CON group did not participate in the training program or undergo any lifestyle modification over the 16 week duration of the study. All subjects volunteered for the study and signed informed consents. TRAIN subjects were compensated for meals, travel time, and training program fees. Exclusion criteria included smoking, known metabolic or cardiovascular conditions (diabetes or dyslipidemia), chronic inflammatory diseases, or chronic use of non-steroidal anti-inflammatory drugs. The protocol was approved by the University of Miami Institutional Review Board for human subjects research.

Initial screening and evaluation included a complete medical and training history, baseline VO_2_ max test, resting heart rate, blood pressure, height, weight, body composition assessment, serum biomarkers, and complete echocardiographic evaluation. Body composition was assessed through dual-energy X-ray absorptiometry scanning. Blood for the lipid panels and serum biomarkers was drawn via the antecubital vein using the LeukoLock Protection system and stored at −80°C for analyses with the post-training RNA samples. Lipid panels were done on fasted blood samples. TRAIN and CON subjects had heart rate, blood pressure, height, weight, and serum biomarkers repeated at 6, 11, and 16 weeks after the baseline evaluation. Echocardiograms, VO_2_ max, and body fat evaluation were recorded at baseline and 16 weeks after the baseline evaluation.

**Figure 1:**
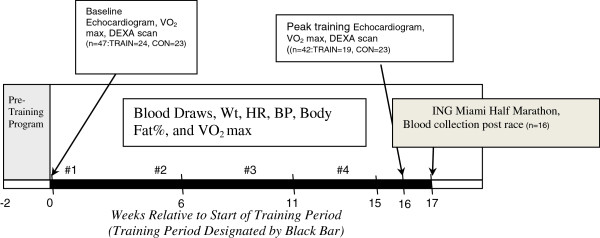
Study design.

Of the 24 subjects enrolled in the TRAIN group, 5 subjects were unable to complete the training due to injury or time constraints and withdrew prior to the peak training data (week 16) collection. Three subjects were able to complete the peak training data collection, but were unable to complete the HM due to injury. Thus, a total of 19 subjects completed the program with peak training data collected. Of these 19 subjects, 16 subjects completed the 2007 Miami HM and had blood drawn within 30 minutes after the race (see Figure 1). Of the CON group, one individual was unable to continue the study as a result of scheduling difficulty, leaving a total of 22 controls that completed all data collection.

Two-dimensional echocardiograms with pulsed-Doppler imaging were acquired on each subject using a commercial system (Philips HD11). Standard measurements were performed according to the American Society of Echocardiography guidelines [11]. Interventricular septal wall thickness, LV internal diameter, and LV posterior wall thickness were measured in systole and diastole in the parasternal long axis view. The LV end-diastolic volume, LV end-systolic volume, stroke volume, and ejection fraction were calculated via modified bi-planer calculation based on the apical 2 and 4 chamber view. LV mass was calculated using the area length method. With the pulsed-Doppler trans-mitral filling velocities (peak early [E’] and late [atrial-A’]), diastolic function was assessed and an E/A ratio was determined. One certified echocardiogram technician was responsible for acquiring all images for each participant. The measurements and calculations were independently confirmed by an experienced echocardiographer. Both were blinded to each subject’s group status.

Blood was collected in EDTA and standard red top tubes (serum) and processed immediately. Tubes were immediately centrifuged, and the serum was separated and stored in a -80° freezer. Serum was analyzed for cTnT [12], CRP [12], and lipids (HDL, LDL, and TG) [12]. Blood for RNA extraction was collected at both baseline and peak training (week 16) using the Ambion LeukoLOCK system that captured and preserved the blood mononuclear cell layer.

Clinical and biomarker data were analyzed using SPSS for Windows 15.0 software (SPSS, Inc, Chicago, IL). Frequency and descriptive statistics were calculated to check all relevant characteristics of the data. Independent samples *t*-tests were used to compare baseline variable differences between the TRAIN and CON groups. Paired samples *t*-tests were used to compare changes from baseline to peak training or baseline to post-HM.

Microarray analysis was performed on blood collected from each subject at baseline and at 16 weeks. Total RNA was extracted and analyzed using the Affymetrix GeneChip Human Gene ST array, which has 28,869 unique genes (or transcripts) which are captured by ~26 probes per gene. Data were normalized using Robust Multi-Array (RMA) Normalization. Significance Analysis of Microarray (SAM) Software [13] was used to determine statistically significant genes that met predetermined False Discovery Rate (FDR) thresholds. SAM uses non-parametric statistics and gene specific *t*-tests to identify statistically significant genes and to measure the strength of relationships between gene expression and a specific response variable. SAM also uses permutation tests to estimate the FDR after accounting for multiple testing corrections [14]. Genes with FDR *p*-values < 0.15 and fold-change greater than 1.5 or less than −1.5 were considered significant. Pathway analysis was conducted using MetaCore™ (GeneGo, Inc) software.

Two separate gene expression analyses were performed. The first compared differences in gene expression profiles between the CON and TRAIN subjects before and after the HM training. The second analysis focused only on the TRAIN subjects, by dividing them into high and low responders based on the percent change of their relative VO_2_ max (rVO_2_ max) over the course of the training program. Relative VO_2_ max takes into account and individual’s body weight and is defined as milliliters of oxygen per kilogram of bodyweight per minute. The mean percent change in rVO_2_ max was calculated and individuals who fell in the upper quartile (upper 25%) of percent change in rVO_2_ max were labeled as high-responders (10 individuals) and those who fell below the upper quartile were considered low-responders (9 individuals). Gene expression differences between the two groups were studied at baseline and after the 16 weeks of training.

## Results

Of the 16 subjects who completed the HM, the average time to completion was 2:22:21 (H:Min:Sec), SD = 26:33. The fastest time was 1:39:00 and the slowest time was 3:11:58. No runner required medical attention after the race. There were no significant differences between the TRAIN and CON groups on any baseline characteristics other than triglycerides, which were significantly higher (p = 0.03) in the TRAIN group (Table 1).

**Table 1:** Training group and control changes at baseline versus 16 weeks (n=22)

	**Training group**			**Control group**			
**Variable**	**Baseline (M±SD)**	**Peak (M±SD)**	P value*	**Baseline (M±SD)**	**Peak (M±SD)**	P value*	P value**
Weight	196.76±28.40	192.85±27.60	0.08	188.31±26.11	190.11±26.41	.064	0.316
Heart rate	76.71±12.41	67.60±11.76	0.002	73.18±12.75	76.05±15.99	.357	0.363
Systolic BP (mmHg)	121.62±8.45	114.30±9.23	0.0001	116.67±9.86	113.36±7.26	.133	0.088
Diastolic BP (mmHg)	80.05±9.70	73.15±8.20	0.007	75.67±8.46	77.14±5.20	.542	0.127
Relative VO_2_ max	37.06±7.12	42.02±8.81	0.00004	38.83±5.03	39.02±6.74	.813	0.35
Body fat (%)	29.51±8.33	28.18±8.86	0.044	26.84±6.84	27.45±6.87	.116	0.256
HDL	46.71±9.61	43.10±6.37	0.055	49.59±7.87	47.05±9.15	.183	0.288
LDL	107.05±25.64	103.80±28.03	0.44	100.23±22.39	105.86±20.61	.087	0.358
Triglycerides	119.90±59.41	116.35±72.01	0.997	85.05±38.84	77.68±40.87	.294	0.03
CRP	1.92±2.66	1.64±1.92	0.752	0.84±0.66	1.03±0.83	.269	0.084
Troponin T	<0.01	<0.01	0.333	<0.01	<0.01	.333	1

*Difference in variable measures between baseline and peak. **Difference at baseline between Training and Control groups.

Comparisons of basic physiological variables were made from baseline to the peak training time-point (17 weeks) in both groups (Table 1). VO_2_ max increased significantly (p = 0.00004) among those in the TRAIN group during the course of the training program. In addition, the following were significantly decreased: (1) resting heart rate (p = 0.002), (2) percent body fat (p = 0.044), and (3) systolic (p = 0.0001) and diastolic (p = 0.007) blood pressure. Average body weight (p = 0.08) and HDL (p = 0.055) decreased slightly (p = 0.08), but no other measured biomarkers (LDL, Tg, CRP, cTnT) showed significant improvements. There were no significant changes for any variables among the CON subjects (Table 1). Table 2 shows comparison of echocardiographic changes between TRAIN and CON groups.

**Table 2:** Echocardiographic changes over 16 weeks (n=19)

	**Training group**			**Control group**		
**Variable**	**Baseline (M±SD)**	**Peak (M±SD)**	**P value**	**Baseline (M±SD)**	**Peak (M±SD)**	**P value**
Interventricular septum in diastole (cm)	0.925±0.102	1.003±0.128	0.028	0.925±0.115	0.982±0.138	0.074
Left ventricular internal diameter in diastole (cm)	4.803±0.490	4.818±0.490	0.864	4.726±0.409	4.876±0.451	0.067
Left ventricular posterior wall in diastole (cm)	0.961±0.092	1.075±0.123	0.003	0.930±0.134	0.963±0.133	0.439
Left ventricular mass (g)	186.6±37.4	209.2±30.0	0.009	183.0±31.1	192.1±32.2	0.114
Left ventricular mass index (g/m^2^)	91.4±16.8	102.8±12.8	0.008	90.4±15.1	94.2±14.1	0.194
Left ventricular end-diastolic volume (ml)	118.4±23.8	144.1±29.6	0.002	124.7±32.7	133.1±30.8	0.186
Left ventricular end-systolic volume (ml)	45.8±16.4	52.4±16.7	0.195	43.4±14.0	48.0±14.8	0.11
Ejection fraction (%)	61.9±7.9	63.8±7.8	0.461	65.2±6.5	64.2±6.5	0.47
Stroke volume (ml)	72.6±14.0	91.6±20.9	0.001	81.2±22.3	85.1±20.4	0.411
Peak early to late mitral annular velocities (E to A ratio)	1.59±0.37	1.38±0.40	0.03	1.67±0.43	1.70±0.48	0.766

Table 1 shows comparison of echocardiographic changes between TRAIN and CON groups. The following significant changes occurred in the TRAIN group: (1) interventricular septal thickness increased from 0.925±0.102 cm to 1.003±0.128 cm (p=0.028), (2) posterior wall thickness in diastole increased from 0.961±0.092 cm to 1.075±0.123 cm (p=0.003), (3) LV mass increased from 186.6±37.4 g to 209.2±30.0 g (p=0.009), (4) LV mass index increased from 91.4±16.8 g/m^2^ to 102.8±12.8 g/m^2^ (p=0.008), (5) LV end diastolic volume increased from 118.4±23.8 ml to 144.1±29.6 ml (p=0.002), (5) stroke volume increased from 72.6±14.0 ml to 91.6±20. 9ml (p=0.001), and (6) the E to A ratio decreased from 1.59±0.37 to 1.38±0.40 (p=0.03). No significant changes occurred within the control group from baseline to 16 week follow-up.

In comparing the gene expression profiles between TRAIN and CON individuals before training, only 4 genes (*CMKLR1, FOS, IFIT1,* and *PDK4*) showed significant changes (FC > 1.5, FDR <0.05) (See Table 3). All had increased levels of gene expression in the TRAIN group compared to the CON group. No genes were significantly decreased.

**Table 3:** Genes with significantly increased expression levels in active (TRAIN) vs. sedentary (CON) individuals (FDR <0.05)

Before marathon training - Increased gene expression*		
**Transcript id**	**Gene**	**Fold change (FC)**
7966089	CMKLR1	1.56
7975779	FOS	1.74
7929065	IFIT1	1.51
8141094	PDK4	1.53
After marathon training - increased gene expression*		
**Transcript id**	**Gene**	**Fold change (FC)**
7929256	---	1.6
7928306	---	1.64
7966089	CMKLR1	1.68
7906475	FCRL6	1.66
7996081	GPR56	1.55
7906764	HSPA6	1.53
7964787	IFNG	1.66
8054722	IL1B	1.71
7961175	KLRC3	1.64
7971661	MIR15A	1.8

*FDR= false discovery rate (p<0.05 threshold used).

When comparing profiles from blood collected in TRAIN and CON individuals after the HM training program, ten genes *(CMKLR1, FCRL6, GPR56, HSPA6, IFNG, IL1B, KLRC3, MIR15A)* showed significantly elevated expression (FC > 1.5, FDR < .05) (Table 3) and 53 genes (including *AREG,DEFA3*) were found to be significantly decreased (FC < −1.5, FDR < .05) in the TRAIN group (Table 4). According to pathway analysis, the biological pathways that were significantly up-regulated in the TRAIN individuals after training (p <0.001) were those involved in inflammatory and immune response, including migration inhibitory factor (MIF) in innate immunity response (p = .00014), bacterial infections in normal airways (p = .0002), apoptosis and survival Nitric Oxide synthesis and signaling (p = .0002) and bacterial infections in CF airways (p = .0002) (data not shown). One biological pathway, translation insulin regulation of translation (p = .00008), showed significantly decreased expression in TRAIN individuals after HM training (data not shown).

**Table 4:** Genes with decreased expression levels in active (TRAIN) vs. sedentary (CON) individuals

**After marathon training - Decreased gene expression**		
**Transcript id**	**Gene**	**Fold change (FC)**
8102787	---	−1.5
8020825	---	−1.7
8037387	---	−1.65
8136471	---	−1.56
8104490	---	−1.56
8084810	---	−1.54
8095744	AREG	−2.42
8095736	AREG	−2.18
8062444	BPI	−1.74
7961075	CD69	−1.69
8149116	DEFA3	−2.42
8149126	DEFA3	−2.42
8149137	DEFA3	−2.34
8084704	EIF4A2	−1.7
8158167	LCN2	−1.51
8086607	LTF	−1.98
7951246	MMP8	−1.95
8012349	PER1	−1.59
7919269	RNU1-1	−1.55
7919349	RNU1-1	−1.55
7973896	RNU1-1	−1.53
7978568	RNU1-1	−1.53
7898375	RNU1-1	−1.52
7898411	RNU1-1	−1.52
7912800	RNU1-1	−1.52
7912850	RNU1-1	−1.52
7919576	RNU1-1	−1.52
7948894	RNU2-1	−1.53
7938295	RPL27A	−1.51
8103975	SLED1	−1.62
7925182	SNORA14B	−1.5
7968234	SNORA27	−1.52
7977075	SNORA28	−1.56
7938293	SNORA45	−1.6
8078918	SNORA62	−1.86
8059708	SNORA75	−2.03
8139456	SNORA9	−1.55
7968232	SNORD102	−1.67
8124940	SNORD117	−1.67
7942592	SNORD15A	−1.53
8059710	SNORD20	−1.93
7903022	SNORD21	−1.69
8159004	SNORD24	−1.83
7948906	SNORD27	−1.69
7948904	SNORD28	−1.51
7948900	SNORD30	−1.8
8030364	SNORD34	−1.64
8159006	SNORD36B	−1.78
8005951	SNORD42B	−1.63
8005957	SNORD4B	−1.63
8023259	SNORD58A	−1.64
7964246	SNORD59B	−1.56
8122265	TNFAIP3	−1.61

The TRAIN group was categorized into 10 high and 9 low responders based on percent change in their rVO_2_ max. Within the TRAIN group alone, 16 genes showed trending evidence of decreased expression (FC > −1.5, FDR < .06) in high vs. low responders, and one gene showed increased gene expression (FC > 1.5, FDR < .06) in the high compared to low responders group (Table 5). Biological processes that were highlighted included those involved in regulation of DNA transcription, translation and other metabolic processes.

**Table 5:** Genes with significant expression in high responders^* ^(n=10) vs. low responders** (n=9)

**After marathon training - Decreased gene expression**		
**Transcript id**	**Gene**	**Fold change (FC)**
7969794	LOC100132099	−1.58
8049530	LRRFIP1	−1.58
7919269	RNU1-1	−1.89
7919349	RNU1-1	−1.89
7898375	RNU1-1	−1.86
7898411	RNU1-1	−1.86
7912800	RNU1-1	−1.86
7912850	RNU1-1	−1.86
7919576	RNU1-1	−1.86
7973896	RNU1-1	−1.85
7978568	RNU1-1	−1.85
7897801	RNU5E	−1.89
7920873	SNORA42	−3.49
8117018	---	−1.86
7932635	---	−1.63
7965150	---	−1.62
**After marathon training - increased gene expression**		
**Transcript id**	**Gene**	**Fold change (FC)**
8005225	LOC162632	2.35

No genes met FDR significance criteria for differential mRNA expression in blood collected at baseline.

*High Responders are defined as those individuals who fell in the upper quartile (upper 25%) of percent change in rVO_2_ max.

**Low Responders are defined as those individuals who fell below the upper quartile of percent change in rVO_2_ max.

## Discussion

This is the first prospective study evaluating the combination of changes in cardiac structure and function, peripheral blood cell gene expression, inflammatory markers, and potential for myocardial injury associated with training for, and performance of, a HM in previously sedentary subjects. The decreases in blood pressure, resting heart rate, and body fat percentage, and the increases in VO_2_ max, observed in our TRAIN group over the course of the program are consistent with previously well-documented responses to exercise training [15,16] and validate the training effect in our HM participants. More interesting is the effect of HM training on biomarkers related to myocardial injury, cardiovascular structural changes by echocardiogram, serum lipids, gene expression and serum inflammatory markers.

In our study, LV mass and wall thickness, and LV end-diastolic and stroke volumes increased significantly after 16 weeks of training. The E to A ratio also decreased throughout training, consistent with a decrease in diastolic relaxation commonly seen in hypertrophied ventricles. These echocardiographic changes have been well-established and recently described to occur in endurance athletes in as little as 90 days [17-19]. This phenomenon, known as the “athlete’s heart,” is thought to be due to a long-sustained high cardiac output resulting in a volume-loaded heart that can lead to an increase in LV cavity, LV mass, and wall thickness [20-22].

Both TRAIN and CON groups had the same inclusion criteria, which enabled us to maximize the comparison after training. When comparing gene expression profiles in blood collected before training in TRAIN versus CON men, 4 genes showed significant changes in gene expression (FC = (1.5-1.7) FDR < .05) suggesting that the two groups were fairly similar before training. Through pathway analysis, the biological pathways that were significantly up regulated in the TRAIN individuals after training were those primarily involved in the body’s inflammatory and immune response. In looking at the specific genes involved of the 53 genes that showed significantly decreased expression in TRAIN individuals after training, Amphiregulin (*AREG*) was most significantly decreased (FC = −2.42). The protein coded by *AREG* is a member of the epidermal growth factor family. *AREG* has both growth factor and inhibitory properties (NCBI, PubMed Gene ID:374). Changes in *AREG* expression in human neutrophils have been related to exercise in previous studies. For example, following 30 minutes of aerobic exercise, *AREG* was shown to increase by 3.4 fold [23]. Although the change is in the opposite direction of the change found in the current study this could be due to the fact that Radom-Aizik et al. [23] examined gene expression changes following acute exercise, whereas our focus is on gene expression changes due to long term, consistent exercise.

Another gene significantly down-regulated in the TRAIN individuals; defensin, alpha 3, neutrophil-specific (*DEFA3*) also has a role in both the immune and inflammatory response (NCBI, PubMed Gene ID: 1668). A previous study linked increased expression of *DEFA3* at both the mRNA and protein level to inflammatory diseases [24]. It is well established that regular exercise helps reduce the amount of total body inflammation and therefore helps prevent the induction of many chronic inflammatory diseases including atherosclerosis. A decrease in total body inflammation is usually associated with a decrease in C-reactive protein, which was not observed in our study. This data suggests that perhaps gene expression changes associated with a decrease in inflammation occur prior to changes in cardiac biomarkers or that subjects with normal CRP at baseline are less likely to show a measurable response to exercise associated with changes in gene expression. Perhaps with longer training, or elevated baseline levels of CPR, similar changes in the cardiac biomarkers would also be seen.

When comparing the TRAIN high responders and low responders, several gene expression differences were noted. Two genes that showed increased expression in high responders before training included *FGFBP2* (FC = 1.76) and *CD160* (FC = 1.55). Both are involved in immune response (NCBI, PubMED Gene ID: 11126).

Of the genes that showed decreased expression in high responders after exercise of particular note is *LRRFIP1* (FC = −1.57). *LRRFIP1* has been identified as having a role as both a regulator of toll-like receptor pathway signaling and transcriptional repression. Its expression has been previously shown to be induced by nicotine and proinflammatory cytokines [25].

## Conclusions

In summary, in previously sedentary individuals, intensive training for a HM over 17 weeks leads to increased cardiac and hemodynamic performance that is not associated with longitudinal changes in known cardiac biomarkers. Elevated cTnT occurred in only 1 of 16 subjects who completed the HM. Training is also associated with changes in blood gene expression that reflects the anabolic state of the body, as well as changes in the body’s immune and inflammatory response that could be more sensitive precursors to changes in serum biomarkers. Whereas some genes involved in the immune/inflammatory response showed increased expression, others showed decreased expression implying that exercise brings about changes that both suppress aspects of the immune system while stimulating others. Genes with changes in gene expression as response to HM-training include *AREG*, *DEFA3*, *CD160*, *GPR56*, *KLRC3*, and *LRRFIP1*. The results presented here are from a single sample and do not withstand conservative corrections for multiple comparisons. The study’s sample is limited to men and therefore cannot necessarily be generalized to the broader population which could include females However, they generate interesting hypotheses about a possible role of genes involved in immunity and inflammation in responding to intensive exercise training, which bear examination in other data sets.

## Competing interests

The authors declare that they have no competing interests.

## Authors’ contributions

DDV: study design, RNA extraction, gene expression and pathway analysis, manuscript preparation. GC: echocardiogram data collection, cardiac biomarker analysis, manuscript preparation. MS: ascertainment, data collection. RJM: study design, cardiac biomarker analysis, manuscript editing. KJ: ascertainment, data collection. LN: microarray and gene expression analysis. AP: data collection, study design, editing. DS: study design, gene expression analysis, manuscript editing. PJGC: study design, manuscript editing. ER statistical analysis, gene expression and pathway analysis, manuscript editing. All authors read and approved the final manuscript.

## Pre-publication history

The pre-publication history for this paper can be accessed here:

http://www.biomedcentral.com/2052-1847/6/6/prepub
